# The Effect of the Ideal Food Pyramid on Gut Microbiota in Rheumatoid Arthritis Patients

**DOI:** 10.3390/life15030463

**Published:** 2025-03-14

**Authors:** Ülger Kaçar Mutlutürk, Betül Çiçek, Gizem Cengiz

**Affiliations:** 1 Department of Nutrition and Dietetics, Institute of Health Sciences, Erciyes University, Kayseri 38039, Türkiye; 2 Department of Nutrition and Dietetics, Faculty of Health Sciences, Erciyes University, Kayseri 38260, Türkiye; bcicek@erciyes.edu.tr; 3 Division of Rheumatology, Department of Physical Medicine and Rehabilitation, Medical Faculty Hospital, Erciyes University, Kayseri 38030, Türkiye; gizemcengiz@erciyes.edu.tr

**Keywords:** gut microbiota, rheumatoid arthritis, ideal food pyramid

## Abstract

Background: The gut microbiota composition of rheumatoid arthritis (RA) patients differs from healthy people, and diet is among the powerful environmental determinants that can alter the microbiota. The purpose of this clinical research was to identify the effect of the Ideal Food Pyramid on gut microbiota in RA, as well as its impact on disease activity, biochemical findings and anthropometric measurements. Methods: Thirty patients diagnosed with RA that met the inclusion criteria were randomized into diet and control groups and followed for 12 weeks. The gut microbiota composition was indicated by 16SrRNA gene sequencing. Results: At the end of this study, Simpson, Shannon and Chao-1 indices were higher in the diet group (16) than in the control group (14), although not significantly (*p* > 0.05). In the diet group, at phylum levels, the abundance of *Bacteroides* decreased while the abundance of *Firmicutes* increased. At species level, *Prevotella copri*, *Bacteroides fragilis*, *Prevotella stercorea*, *Bacteroides uniformis* decreased, while *Faecalibacterium prausnitzii*, *Roseburia faecis*, *Bacteroides ovatus*, *Akkermansia muciniphila*, *Coprococcus eutactus*, *Gemmiger formicilis*, *Ruminococcus bromii*, and *Bifidobacterium longum* species increased in the diet group. Conclusions: The Ideal Food Pyramid has been determined to have many clinical benefits for RA patients, especially for the gut microbiota.

## 1. Introduction

RA is a common systemic inflammatory autoimmune disease that causes synovium hyperplasia and bone–joint destruction [1]. RA is guessed to influence nearly 0.24 to 1 per cent of the population and is more widespread among females than males [2]. RA is a form of arthritis that gives rise to swelling, pain, stiffness and function loss in the articulars. This disease seriously affects quality of life with increasing morbidity and mortality [3]. Interactions among genetic factors, environmental factors and the existence of autoantibodies cause an escalated risk of growing RA [4]. In the previous years, developing verity has indicated that gut dysbiosis plays a role in the beginning autoimmune diseases as RA, proposing its role in contributing to impaired immune tolerance [5,6]. Filamentous bacteria in the gut have been reported to trigger autoimmune arthritis through their effects on T helper 17 (Th17) cells [7]. Increasing evidence suggests that microbiome dysbiosis may disrupt gut barrier integrity, including the mucus layer and epithelial cell junctions, leading to increased gut permeability [8,9]. This situation fuels host immune responses and chronic inflammatory disorders by allowing the translocation of harmful microbiome-derived and environmental constituents into the mucosal segment and systemic circulation [9]. Patients with RA show significant differences in the composition of the intestine microbiota compared with controls. The gut microbiome in individuals with RA has shown an increased abundance of *Prevotella* (especially *Prevotella copri*), *Collinsella aerofaciens* and decreased abundance of *Faecalibacterium prausnitzii*, *Bacteroides fragilis* [5,10]. Although the mechanism of how *Prevotella copri* promotes the progress of RA is still unclear, the high amount of *Prevotella copri* in the gut in patients with RA exacerbates the humoral immune response. Animal studies have demonstrated Th1 cell activity against *Prevotella copri*, which in turn induces autoreactive T cell activation in mice. In addition, experiments have identified the up-regulation of Th17-related cytokines such as IL-6 and IL-23 by *Prevotella copri* [10]. In addition to supporting disease activity, gut microbiota may also affect the response of patients with RA to treatment [11]. Therefore, gut microbiota constitutes a potential therapeutic target in patients with RA, both in terms of regulating disease and improving response to current therapies. The literature suggests that diet plays an important regulatory role in RA because it is an environmental factor that affects antigen production, inflammation, antioxidant defence mechanisms and gut microbiota [12,13]. One of the strongest ways to change gut microbiota is through diet [14]. One of the contraptions of anti or pro-inflammatory action of the diet has a medioxumous effect on the metabolic activity and composition of the intestinal microbiota. The intestinal microbiota is incredibly dynamic and responds rapidly to dietary changes [8]. High-fibre diets (e.g., Mediterranean diet, vegan, vegetarian) are associated with increased microbial diversity and a healthy microbiome composition with more beneficial bacteria (e.g., *Bifidobacteria, Eubacterium*, *Roseburia*, *Lactobacillus*). In addition, they are connected with higher levels of short-chain fatty acids (SCFAs) [13]. In contrast, Western diets high in protein and animal fat and low in fibre increase pathogenic bacteria (e.g., Proteobacteria, *Collinsella*, *Bacteroides*, *Enterobacteria*) while decreasing beneficial bacteria (e.g., *Eubacterium, Bifidobacteria* and *Lactobacillus*) with an overall decrease in total bacterial abundance [13]. However, the available evidence on diet and RA is insufficient, and evidence-based nutritional guidelines for the administration of RA are currently lacking. Although the importance of the Mediterranean diet pyramid recommended for the general population in autoimmune diseases is emphasized, the results are contradictory and insufficient [15,16,17]. Recently, Rondanelli et al. recently proposed an adapted food pyramid for patients with RA based on the principles of the Mediterranean diet [12]. This pyramid has important differences from the Mediterranean diet:

1. The base contains five portions of vegetables and fruits (indicating the most beneficial types of vegetables) instead of carbohydrates;
2. Preferably gluten-free cereal consumption;
3. Daily consumption of seeds (flaxseed, chia seeds);
4. Two flags are added to the top of the pyramid, emphasizing that RA patients should avoid salt and simple sugar intake and that it is beneficial to take omega 3, vitamin D and antioxidant supplements [12]. This pyramid provides bioactive nutrients, including fibre, antioxidants, polyphenols, vitamins, minerals, and omega-3 polyunsaturated fats, many of which may promote beneficial health effects via the gut microbiota. This research claims to determine the effects of an Ideal Food Pyramid specifically designed for RA on disease activity, biochemical analyses, body composition and gut microbiota.

## 2. Materials and Methods

### 2.1. Clinical Trial Design

This research was conducted as a single centre, randomized-controlled, open label, clinical trial in the Rheumatology Outpatient Clinic of Erciyes University Faculty of Medicine Hospital between May 2023 and January 2024. Power analysis calculation was not performed at the beginning of this study. According to the European League Against Rheumatism EULAR)/American College of Rheumatology (ACR) classification criteria [18], 30 patients (14 control and 16 diet) who were diagnosed with RA and met the inclusion criteria were involved in this research, and a post-study power analysis was carried out. The results regarding the comparison of *Prevotella* levels between the study groups both at baseline and end-of-trial intervention were statistically significant (*p* < 0.001). Post-test power values for type-I error probability of 5% were obtained as 100% both before and after the intervention. Since these values were above 94%, it was decided to terminate this study with the current sample size. The Ideal Food Pyramid specially prepared for RA was explained to the diet group, and a nutrition programme suitable for the pyramid was created. Participants in the control group were asked to maintain their usual diet. Both groups were followed up for 12 weeks. Fecal samples, blood findings, anthropometric measurements and disease activity were collected at 0 and 12 weeks and compared.

Consent declaration information form was obtained from the study participants. Ethical approval of this study was approved by Erciyes University Faculty of Medicine Clinical Research Ethics Committee on 23 February 2022 (Approval Number: 2022/148). This study is registered at ClinicalTrials.gov ID: NCT06621927. Release Date: 27 September 2024.

### 2.2. Participants

Inclusion criteria

- Being diagnosed with RA by a rheumatologist and starting disease-modifying antirheumatic drug (DMARD) treatment.
- RA disease duration longer than 1 year.
- Age range 18–65.
- Body mass index (BMI) = 18.5–40 kg/m^2^.
- Smoking three and less than three cigarettes a day.

Exclusion criteria

- Diabetes, cancer, inflammatory bowel disease, kidney and liver disease and psychiatric disorders.
- Use biological drugs, regular users of Non-Steroidal Anti-inflammatory Drugs (NSAIDs) and oral cortisol intake > 12.5 mg.
- Those who have used a special diet, herbal supplements, vitamin-mineral supplements (except D vit.) and probiotics in the last 3 months.
- Those who received antibiotic treatment in the last 3 months.
- Pregnant or lactating women.

Patients were involved in this research through a volunteer foundation and signed an acknowledged consent form.

### 2.3. Dietary Intervention

An individual specific nutrition programme was prepared for the diet group in accordance with the Ideal Food Pyramid prepared by Rondanelli et al. [12]. According to the pyramid, daily consumption of 5 portions of vegetables and fruit was recommended. Especially cruciferous vegetables and green leafy vegetables, red fruits and citrus fruits were recommended. Cereals were recommended as 3 servings of whole grains per day, preferably gluten-free. Sugar and sweeteners were excluded from the diet. Extra virgin olive oil (10 g), omega-3 rich flax or chia seeds (10 g), nuts (30 g), spices such as turmeric, ginger, chilli, cinnamon, low-fat yoghurt (125 g) and low fat milk (150 g) were among the daily recommendations. Although daily drinking of red wine (125 g) was among the recommendations, this recommendation was not made since there was no alcohol consumption among the patients. On a weekly basis, 3 portions of fish, white meat and legumes, preferably fatty, 2 portions of eggs and cheese per week, and 1 portion of red or processed meat per week were recommended. There were two flags at the top of the pyramid: the green flag indicates that taking vitamin D, omega 3 and antioxidant supplements may be beneficial, while the red flag indicates that salt and sugar intake is prohibited. In the diet group, a telephone call was provided by a dietitian every 15 days to increase dietary adherence. The phone call provided participants with the opportunity to ask questions about the diet plan (such as preparation, cooking) and to report any negative effects. No diet application was mentioned to the control group. They were told to continue their usual diet.

### 2.4. DataCollection

#### 2.4.1. Anthropometric Measurements

Body weight (kg), lean body mass (kg) and body fat percentage (%) were measured using Tanita BC 418MA brand (bioelectrical impedance analysis [BIA]). BMI = Weight (kg)/Height (m^2^) formula was calculated. For BMI measuring, individuals were required not to perform heavy physical activity 24–48 h before, not to drink alcohol 24 h before, to come with at least 4 h of fasting, not to consume too much liquid (water, tea, coffee) before the analysis (at least 4 h) and not to keep metal objects on them. Waist, hip, wrist and neck circumference and height measurements were taken by the researchers using a non-stretchable plastic tape measure in accordance with the technique. Hand grip strength was measured to obtain information about the muscle development of the patients. Jamar hand dynamometer, recommended by the American Association of Hand-Therapists (AETD) and accepted as the gold standard, was used to measure hand grip strength. Patients were measured while sitting, positioned in shoulder adduction and neutral rotation, forearm in mid-rotation and supported, elbow in 90-degree flexion, wrist in neutral position, each hand was measured 3 times and the average of 3 measurements was taken [19].

#### 2.4.2. Disease Activity

Firstly, the tender and swollen joints of all patients were identified by physical examination by a rheumatologist. The composite scores DAS28-ESR and DAS28-CRP involve the number of swollen and tender joints in 28 joints, with the patient’s estimate of their general health on the VAS, and ESR or CRP [20]. The SDAI is calculated as the numerical sum of tender and swollen joint patient general assessment, physician general assessment and CRP level (mg/dL) [21].

#### 2.4.3. Biochemical Parameters

After overnight fasting, blood samples were taken at the baseline and end of trial. Analyses were performed in the laboratory of Erciyes University Medical Faculty Hospital. Fasting plasma glucose (FPG), total cholesterol, triglyceride, high-density lipoprotein (HDL), low-density lipoprotein (LDL), cholesterol, cholesterol, urea, uric acid, creatinine, alanine aminotransferase (ALT) and aspartate aminotransferase (AST) were measured by spectrophotometric method in Cobas c701 (Roche Diagnostic, Basel, Switzerland) analyzer. CRP was measured by immunoturbidimetric method in Cobos c701 (Roche Diagnostic, Basel, Switzerland) analyzer. Sedimentation rate was measured by Vision C ESR analyzer (YHLO, Shenzhen, China).

#### 2.4.4. Fecal Sampling and 16S-Ribosomal-RNA Gene Sequencing

Fecal samples were collected from all participants at the baseline and end of trial during this study and stored at −80 °C for further processing. After sample collection was completed, Deoxyribonucleic Acid (DNA) isolations were performed. DNA extraction from fecal samples was performed using the Qiagen Power Soil DNA Extraction Kit (Qiagen, Hilden, Germany). Quantification of double-stranded DNA (dsDNA) was performed using the Qubit dsDNA HS Assay Kit and a Qubit 2.0 Fluorimeter (Thermo Fisher Scientific, Waltham, MA, USA). After dsDNA measurements, extracted DNA samples were stored at −20 °C until needed for subsequent analysis. 16S-ribosomal-RNA sequencing (rRNA) was performed using the “16S Metagenomic Sequencing Library Preparation: Preparation of 16S rRNA Gene Amplicons for the Illumina MiSeq System”, which was applied on an Illumina MiSeq system (Illumina-San Diego, CA, USA) following the manufacturer’s protocol for “Preparation of 16S Ribosomal RNA Gene Amplicons for an Illumina MiS MiSeq System”. To construct the sequencing library, V4 hypervariable regions were amplified using primer sets 515F (5′--GTGCCAGCMGCCGCGGTAA---3′) and 816R (5′ GGACTACHVGGGTWTCTAAT-3′) [22]. For final sequencing library preparation, 15% PhiXControl (v3) (Illumina, San Diego, CA, USA) was introduced into the library. The libraries were then subjected to cluster generation and sequencing in 250PE-MiSeq runs generating at least 50,000 reads per sample.

#### 2.4.5. Bioinformatic Analysis

Paired-end Illumina reads (2 × 250) were sent to qiime2 environment [23]. Quality clipping, chimera detection, and cleaning of reads implemented through the Qiime2Dada2 pipeline (via q2dada2) [24] and bases that have low Phred score (<Q30) were picked out. Amplicon Sequence Variants (ASVs) generated by Dada2 were processed into the database [25,26]. The Phyloseq [26] object was designed from qiime2 artefact files in the R4.1 environment [27,28]. Alpha diversity, used to assess the diversity of related taxonomic units in a sample, is shown using three different indices including Chao1, Shannon and Simpson. *p* values between groups were calculated using the Kruskal–Wallis test [29]. Beta diversity analysis was performed to assess taxonomic differences among individuals and calculated using weighted unifrac, unweighted unifrac, Bray–Curtis and Jaccard. Statistical significance of beta diversity between groups was established using the PERMANOVA test with the Adonis function in the vegan R package. Specific differences between groups were determined by differential abundance analysis using the Deseq2 R package [30].

#### 2.4.6. Statistical Analysis

Shapiro–Wilk test, histogram and q-q graphs were used to evaluate data normality. Arithmetic mean and standard deviation, median (25th–75th percentile), percentage and frequency statistics were used to summarize the data. Independent Mann–Whitney U test and two-sample *t*-test were used for intergroup comparisons of quantitative data. Dependent Wilcoxon test and two-sample *t*-test were used to compare baseline and end of trial parameters. Fisher’s exact chi-square test was used to compare categorical data. Data were analyzed using TURCOSA (TurcosaAnalytics, Ltd. Co., Kayseri Turkey, www.turcosa.com.tr, accessed on 10 June 2024) statistical software. Power analyses were performed with SigmaStat 3.5 statistical software (Systat Software Inc., San Jose, CA, USA, https://sigmastat.software.informer.com/ accessed on 20 June 2024). In all statistical analyses, the significance level was accepted as *p* < 0.05, and the confidence interval was 95%.

## 3. Results

A total of 30 randomly selected participants (diet 16, control 14) completed the research. The mean age of the control group was 53.71 ± 7.36 years, while the mean age of the diet group was 49.25 ± 10.44 years. The majority of the participants were women (86.7%). No significant differences were seen between groups in demographic characteristics at baseline (*p* > 0.005) (Table 1).

### 3.1. Anthropometric Measurements

At the end of the trial, when the diet group (*n* = 16) was compared with the control group (*n* = 14), significant improvements were recorded in body composition (body weight (*p* = 0.002), body mass index (BMI) (*p* = 0.015), fat percentage (*p* = 0.014), fat mass (*p* = 0.008), waist circumference (*p* < 0.001), waist/height ratio (*p* = 0.000), hip circumference (*p* < 0.001), waist/hip ratio (*p* = 0.031), neck circumference (*p* = 0.001), wrist circumference (*p* = 0.001), height/wrist circumference (*p* = 0.001) and hand grip strength (*p* < 0.001) (Table 2).

### 3.2. Biochemical Parameters

At the end of trial, there were significant differences in CRP (*p* < 0.001), ESR (*p* < 0.001), LDL cholesterol (*p* = 0.013) and total cholesterol (*p* = 0.008) values between the diet and control groups. Uric acid decreased significantly in the diet group at the end of the trial (*p* = 0.038) (Table 3).

### 3.3. Disease Activity

At the end of trial dietary intervention, the number of tender joints decreased significantly (*p* < 0.001). DAS28-ESR, DAS28-CRP and SDAI, which are scales used to determine the activity score of RA disease, decreased significantly in the diet group compared to baseline (*p* < 0.00, *p* < 0.001, *p* = 0.008, respectively) (Table 4).

### 3.4. Fecal Microbiota Composition

**Figure 1:**
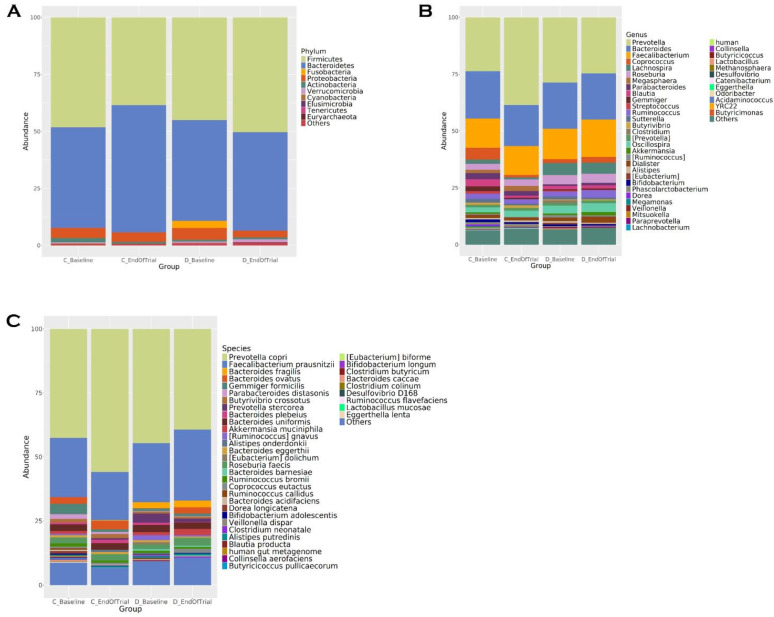
Intestinal microbiota composition of the study groups at phylum, species and genus levels. Bacterial community relative abundance analysis at the phylum (**A**), genus (**B**) and species (**C**) levels (relative abundance > 1%; bacteria with relative abundances < 1% are pooled in the “others” category and sorted by total concentration.

At the phylum level, in the diet group, at the end of 12 weeks, the abundances of *Firmicutes* (45.184–50.916%), *Actinobacteria* (0.927–0.996%), *Verrucomicrobia* (0.434–1.341%), *Elusimicrobia* (0.002–0.238%) and *Euryarchaeota* (0.004–0.119%) increased, while the abundances of *Bacteroidetes* (43.43–42.682%), *Proteobacteria* (5.923–3.011%), *Fusobacteria* (3.251–0.009%), *Cyanobacteria* (0.35–0.234%) and *Tenericutes* (0.401–0.328%) decreased. In the control group, *Firmicutes* (47.651–38.633%*)*, *Proteobacteria* (4.393–4.231%), *Actinobacteria* (1.723–1.137%) and *Cyanobacteria* (0.407–0.07%) decreased, while *Bacteroidetes* (44.988–55.63%) and *Verrucomicrobia* (0.605–0.173%) increased (Figure 1A).

Microbiota composition is shown in Figure 1B,C according to genus and species levels. At the end of the trial, in the diet group, the genus levels of *Prevotella* (28.134–25.234%), *Bacteroides* (20.806–19.511%), *Lachnospira* (5.635–5.001%), *Clostridium* (2.021–0.54%), *Blautia* (1.976–1.573%), *Sutterella* (1.494–0.896%), *Stretococcus* (0.594–0.338%), *Lactobacillus* (0.233–0.043%) and *Collinsella* (0.242–0.141%) decreased in abundance; *Faecalibacterium* (13.023–16.844%), *Roseburia* (4.002–4.615%), *Oscillospira* (3.406–4.061%), *Ruminococcus* (2.185–3.519%), *Akkermansia* (0.547–1.61%), *Coprococcus* (1.888–2.887%), *Parabacteroides* (0.919–1.457%) and *Gemmiger* (0.988–1.136%), increased. In the control group, at the end of trial, *Prevotella* (25.783–38.719%), *Megasphaera* (1.185–2.513%), *Alistipes* (0.85–1.254%) and *Oscillospira* (2.63–2.879%) relative abundances increased, while *Bacteroides* (19.953–17.972%), *Faecalibacterium* (13.209–12.759%), *Coprococcus* (5.452–1.587%), *Parabacteroides* (2.97–2.23%), *Blautia* (3.082–1.172%), *Gemmiger* (2.182–0.972%), *Ruminococcus* (2.405–2.253%), *Sutterella* (1.813–1.258%), *Bifidobacterium* (1.258–1.016%), *Lachnospira* (2.04–1.199%), *Dorea* (1.102–0.395%) and *Akkermansia* (0.732–0.166%) relative abundances decreased.

At the species level, in the diet group, *Prevotella copri* (44.112–39.508%), *Bacteroides fragilis* (3.494–2.539%), *Prevotella stercorea* (3.582–1.621%), *Bacteroides uniformis* (3.114–2.962%), *[Ruminococcus] gnavus* (2.293–0.611%) and *[Eubacterium] dolichum* (1.145–0.01%) species decreased; *Faecalibacterium prausnitzii* (22.419–28.151%), *Roseburia faecis* (2.322–3.611%), *Bacteroides ovatus* (0.795–2.84%) and *Akkermansia muciniphila* (0.947–2.71%) increased. In the control group, at the end of trial, *Prevotella Copri* (45.02–56.228%), *Bacteroides ovatus* (2.915–3.755%) and *Roseburia faecis* (2.497–2.869%) relative abundance of its species increases; *Faecalibacterium prausnitzii* (23.153–18.752%), *Gemmiger formicilis* (3.856–1.435%), *Bacteroides uniformis* (2.905–2.838%), *Parabacteroides distasonis* (2.326–1.212%), *Ruminococcus bromii* (1.439–1.241%) and *Akkermansia muciniphila* (1.293–0.244%) species have decreased in relative abundance. A bar plot representation of family, order and class levels of bacteria are given in the Supplementary Material (Figure S1, Figure S2, Figure S3 respectively).

### 3.5. Alpha–Beta Diversity

**Figure 2:**
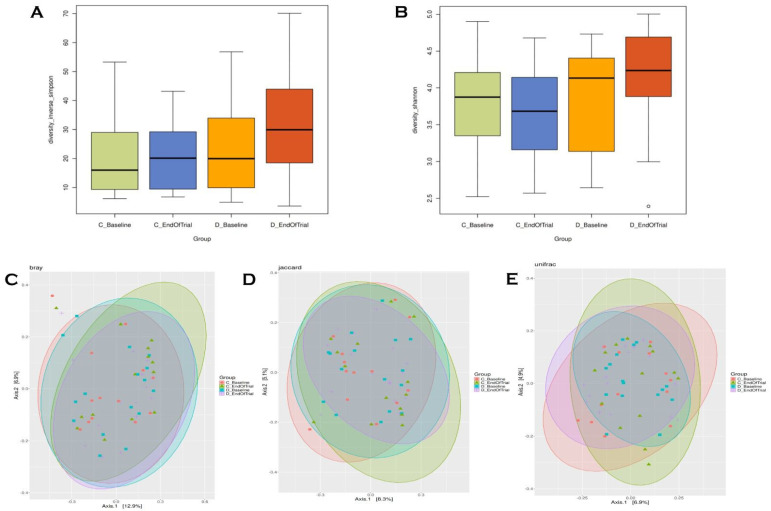
Alpha diversity analysis and beta diversity analysis. Simpson index (**A**) and Shannon index (**B**) plotted by the treatment group and time point. The box spans the first and third quartiles. A horizontal line marks the median, and the whiskers represent ±1.5 times the interquartile range. The outlier (panel **B**) is marked as an individual point. (**C**–**E**) shows PCoA 2D plots of beta diversity analysis of diet (baseline−end of trial) and control groups (baseline–end of trial). Each dot represents a fecal sample. The red circle, green triangle, blue square and purple plus represent control_baseline, control_post, diet_baseline and post_diet, respectively. (**C**–**E**) differences between samples are measured by Bray–Curtis distances (**C**), Jaccard distance (**D**) and unweighted UniFrac distances (**E**). The groups show clustering in similar areas in Bray–Curtis (**C**), Jaccard (**D**) and unweighted UniFrac (**E**) baseline coordinate analysis (PCoA) results.

Although the median of the Amplicon Sequence Variant (ASV) value in the fecal samples taken at the end of trial in the diet group was higher than baseline, no significant difference was found in the Simpson and Shannon index of ASV levels (*p* = 0.26, *p* = 0.51, respectively). Simpson is an alpha measurement expressing the diversity of species. In the control group, the species distribution in the 25th–75th percentile ranges is similar in the baseline and end-of-trial fecal sample data (*p* = 0.92) (Figure 2A). The Shannon index is a measure of uniformity and richness that takes entropy into account. At the end of trial, the Shannon index increased in the diet group (*p* = 0.51) and decreased in the control group (*p* = 0.98) (Figure 2B). According to Bray–Curtis (Figure 2C), Jaccard (Figure 2D) and unweighted UniFrac (Figure 2E) principal coordinate analysis (PCoA) results, there is no statistically significant difference in microbial diversity between or within the control and diet group at baseline and at the end of trial.

Although there was no significant result in alpha and beta diversity, there was a difference between the diet and control groups as seen in the graphs. While *Prevotella copri*, which had increased abundance in RA and decreased in the diet group, the relative abundances of benign *Faecalibacterium prausnitzii*, *Roseburia faecis*, *Bacteroides ovatus* and *Akkermansia muciniphila*, which had decreased abundance in RA, increased.

## 4. Discussion

Our study is the first to apply the Ideal Food Pyramid for RA. In our study, we first showed that the composition of the gut microbiota in the RA patient group who followed the diet was different from the control group. These findings suggest that the gut microbiota composition may be affected by dietary intervention. In addition, the ideal food pyramid created for RA patients has shown positive effects on anthropometric measurements, disease activity and some biochemical markers. Diet has received significant attention as a potential environmental factor influencing the prognosis of RA [31]. Research data strongly suggest that diets with anti-inflammatory properties, such as the Mediterranean diet, which is rich in fish, vegetables, and fruits, may help improve symptoms and/or delay the onset of RA. Therefore, nutrition should be routinely addressed to facilitate disease management in RA [15,32]. Given that various abnormalities have been identified in the intestinal microbiota of RA patients and that a healthy diet can influence gut dysbiosis, diet is an important part of RA disease treatment [33]. The production of SCFA is one of the mechanisms by which the gut microbiota has been proposed to influence the differentiation of Treg cells and systemic inflammation [34]. SCFAs are a source of energy for intestinal epithelial cells, which have an indirect anti-inflammatory effect by enhancing gut barrier function and improving tight junction assembly. SCFAs, especially acetate, butyrate and propionate, are essential metabolites arising from the microbial fermentation of dietary fibres [34]. The positive effects of the Ideal Food Pyramid on microbiota may be due to its high content of dietary fibre, polyphenols and omega-3 fatty acids. The Ideal Food Pyramid rich in fibre includes vegetables, fruits, seeds, nuts and legumes, which are ‘microbiota-accessible carbohydrates’ (MACs). MACs may support the growth of SCFA-producing species and alter the gut microbiota [35]. In addition to dietary fibre, most polyphenols reach the intestine and are either absorbed or undergo gastrointestinal biotransformation in the intestine. Polyphenols exert localized interactions with the microbiota and affect the immune status of the intestinal epithelial layer [36]. Omega-3 fatty acids also encourage the production and secretion of intestinal alkaline phosphatase, which leads to changes in the intestinal microbiota. In addition, omega-3 fatty acids reduce inflammation and endotoxemia by reducing lipopolysaccharide production and intestinal permeability [37]. Although the literature is not consistent, there is a lot of evidence linking RA to dysbiotic gut disorders. However, there is a common finding across studies; there is an increase in the genus *Prevotella*, and particularly the species *Prevotella copri*, in patients with early RA [38,39]. Chen et al. found high amounts of *Prevotella copri* in fecal samples from patients with recent-onset RA [10]. Similarly, Seifert et al. showed that RA patients had higher serum levels of anti-Pcp27 IgA and anti-Pcp27 IgG antibodies (immunogenic *P. copri* 27 kDa protein) than controls [40]. The mechanisms suggested to contribute to the pathogenesis of RA by *Prevotella copri* include the induction of inflammatory responses and molecular mimicry [6]. In our present study, in accordance with these data, *Prevotella* genus and *Prevotella copri* species, which were at high levels at the beginning, decreased with dietary intervention. In the control group, both increased.

*Faecalibacterium* and *Roseburia* are reduced in RA and other inflammatory diseases [33,41]. Bacteria belonging to these genera are well-known butyrate producers, help maintain the integrity and health of the intestinal epithelial barrier and exhibit anti-inflammatory properties [41]. In addition, *Faecalibacterium prausnitzii (F. prausnitzii)*, a species of the *Faecalibacterium* genus that is a member of the human commensal gut microbiota with reduced abundance in RA patients, has been shown to maintain Th17/Treg balance and intestinal barrier function and also exhibit significant anti-inflammatory effects [42]. *F. prausnitzii* reduces the arthritis score, joint tissue damage, and the number of systemic immune cells secreting IL17. Also, it affects the composition of the gut microbiome and causes changes in SCFA concentrations. Therefore, *F. prausnitzii* may have a potential therapeutic effect in RA [43]. Although studies on microbiota in RA are limited, most of them are animal studies. In addition, the number of studies on the effect of nutrition and diet on microbiota is very few. In a recent literature review, it was determined that dietary elements such as fibres, polysaccharides, resistant starch and peptides given to experimental animals were effective in the fight against RA [44].

When analyzing human studies, Peltonen et al. [45] found a significant change in gut flora after a one-year transition from a traditional diet to a vegan and then lacto-vegetarian diet in a study of 53 RA patients. They also noted a significant difference in the microbiota composition of test subjects in the low- and high-improvement groups, suggesting a link between gut microbiota and disease activity levels. A 2005 study reported that the Mediterranean diet and fasting did not affect gut microbiota in RA patients [16]. A recent study showed that RA patients with high adherence to the Mediterranean diet had a healthier gut microbiota. An almost complete absence of *P. copri* was observed in the high adherence group compared to the low/medium adherence group [46].

As far as we know, this comprehensive study is the first to investigate the protective effects of the Ideal Food Pyramid for RA patients on disease activity, biochemical parameters, anthropometric measurements and gut microbiota in RA patients. This protective effect is probably due to the RA-specific recommendations of the pyramid (anti-inflammatory, antioxidant, prebiotic content). Personalized nutrition and dietary guidance may improve diet quality and disease management.

Although this pyramid is similar to the Mediterranean pyramid recommended for the general population, there are important differences. The base of the pyramid emphasizes the consumption of vegetables and fruit instead of carbohydrates (with particular reference to the types to be eaten) and carbohydrates preferably gluten-free. At the top of the pyramid is a red flag indicating that salt and sugar should be avoided and a green flag indicating that vitamin D, omega-3 and antioxidant supplements are beneficial. Our study has some limitations. The lack of blinding represents a potential bias that needs to be taken into account. Dietary intervention studies are difficult to implement and follow up. Although follow-up is performed, it is also difficult to ensure compliance with the diet. Perhaps a compliance scale could be created to assess dietary adherence.

## 5. Conclusions

In conclusion, the Ideal Food Pyramid applied to RA patients for 12 weeks has positive effects on inflammation, lipid profiles, anthropometric measurements, disease activities, and especially on the gut microbiota. The evidence obtained from this study highlights the promising role of disease-specific tailored dietary intervention in regulating the gut microbiota as a complementary approach to traditional RA treatments. Focusing diet on improving intestinal barrier function and altering dysbiosis in the microbiota “may become a valuable part of preventive nutritional goals. This is a valuable study as it is the first study to demonstrate the effect of this pyramid specifically created for RA on the microbiota. In the future, randomized clinical trials with larger sample sizes using the Ideal Food Pyramid in RA are needed to confirm our results.

### Limitations

RA is more common in females than in males, so the number of females in our study was higher, and the female/male ratio could not be equal. Additionally, microbiota studies are expensive, so we could not further increase the number of participants. 

## Figures and Tables

**Table 1:** Patients’ characteristics.

Variables	Control (*n* = 14)	Diet (*n* = 16)	Total (*n* = 30)	*p*
**Age**	53.71 ± 7.36	49.25 ± 10.44	51.33 ± 9.26	0.193
**Sex**				1.000
**Female**	12 (85.7%)	14 (87.5%)	26 (86.7%)	
**Male**	2 (14.3%)	2 (12.5%)	4 (13.3%)	
**Place of residence**				1.000
**Urban**	13 (92.9%)	15 (93.7%)	28 (93.3%)	
**Rural**	1 (7.1%)	1 (6.3%)	2 (6.7%)	
**Marital status**				0.814
**Married**	11 (78.6%)	13 (81.2%)	24 (80.0%)	
**Single**	0 (0%)	1 (6.3%)	1 (3.3%)	
**Widow**	3 (21.4%)	2 (12.5%)	5 (16.7%)	
**Level of education**				0.200
**Primary school**	7 (50.0%)	8 (50.0%)	15 (50.0%)	
**Middle school**	3 (21.4%)	0 (0%)	3 (10.0%)	
**High school**	4 (28.6%)	6 (37.5%)	10 (33.3%)	
**University**	0 (0%)	2 (12.5%)	2 (6.7%)	
**Employment**				0.814
**Housewife**	11 (78.6%)	13 (81.3%)	24 (80.0%)	
**Full-time job**	2 (14.3%)	3 (18.8%)	5 (16.7%)	
**Retired**	1 (7.1%)	0 (0%)	1 (3.3%)	
**Socioeconomic status**				0.840
**Low**	5 (35.7%)	4 (25.0%)	9 (30.0%)	
**Medium**	9 (64.3%)	11 (68.8%)	20 (66.7%)	
**High**	0 (0%)	1 (6.3%)	1 (3.3%)	
**Mode of delivery**				
**Vaginal birth**	14 (100.0%)	16 (100.0%)	30 (100.0%)	
**Cesarean section**	0 (0%)	0 (0%)	0 (0%)	
**Duration of breastfeeding (months)**	8.00 ± 9.98	13.40 ± 7.72	11.38 ± 8.73	
**Physical activity**				0.440
**Inactive**	11 (78.6%)	10 (62.5%)	21 (70.0%)	
**Minimal active**	3 (21.4%)	6 (37.5%)	9 (30.0%)	
**Active**	0 (0%)	0 (0%)	0 (0%)	
**Gingivitis**	1 (7.1%)	2 (12.5%)	3 (10.0%)	1.000
**RA disease duration (Years)**	13.21 ± 6.69	11.56 ± 8.49	12.33 ± 7.62	0.563
**Sleep duration (hours)**	6.57 ± 1.28	7.13 ± 1.26	6.87 ± 1.28	0.244

Data are expressed as percentage (%) or mean ± standard deviation.

**Table 2:** Anthropometric measurements.

Parameters	Control (*n* = 14)	Diet (*n* = 16)	*p*
**Body weight (kg)**			
**Baseline**	79.75 ± 12.80	75.26 ± 13.52	
**End of trial**	80.96 ± 13.14	73.81 ± 13.49	
***p* ^‡^**	0.009	0.020	
**Change**	−1.20 ± 1.46	1.45 ± 2.22	0.001
**BMI (kg/m^2^)**			
**Baseline**	31.44 ± 6.06	29.18 ± 4.90	
**End of trial**	31.93 ± 6.31	28.57 ± 4.69	
***p* ^‡^**	0.012	0.015	
**Change**	−0.49 ± 0.62	0.61 ± 0.89	0.001
**Percent fat (%)**			
**Baseline**	37.04 ± 7.24	35.74 ± 7.81	
**End of trial**	42.91 ± 10.20	33.70 ± 7.99	
***p* ^‡^**	0.001	0.014	
**Change**	−5.87 ± 5.31	2.04 ± 2.93	0.000
**Fat mass (kg)**			
**Baseline**	30.12 ± 9.49	27.33 ± 9.87	
**End of trial**	35.51 ± 13.08	25.48 ± 9.92	
***p* ^‡^**	0.003	0.008	
**Change**	−5.39 ± 5.46	1.86 ± 2.42	0.000
**Muscle mass (kg)**			
**Baseline**	49.65 ± 6.19	47.84 ± 6.27	
**End of trial**	45.39 ± 6.19	48.83 ± 7.21	
***p* ^‡^**	0.008	0.026	
**Change**	4.26 ± 5.12	−0.99 ± 1.60	0.002
**Waist circumference (cm)**			
**Baseline**	105.57 ± 8.34	99.75 ± 12.22	
**End of trial**	109.71 ± 9.16	95.13 ± 10.24	
***p* ^‡^**	0.000	0.000	
**Change**	−4.14 ± 2.35	4.63 ± 3.40	0.000
**Hip circumference (cm)**			
**Baseline**	119.07 ± 11.45	114.63 ± 8.71	
**End of trial**	119.93 ± 11.81	111.75 ± 8.73	
***p*** **^‡^**	0.047	0.000	
**Change**	−0.86 ± 1.46	2.88 ± 2.39	0.000
**Waist/Hip ratio**			
**Baseline**	0.89 ± 0.04	0.87 ± 0.07	
**End of trial**	0.93 ± 0.05	0.85 ± 0.06	
***p* ^‡^**	0.010	0.031	
**Change**	−0.14 ± 0.05	0.02 ± 0.03	0.001
**Waist/height ratio**			
**Baseline**	0.66 ± 0.07	0.62 ± 0.08	
**End of trial**	0.69 ± 0.08	0.59 ± 0.07	
***p* ^‡^**	0.000	0.000	0.000
**Change**	−0.03 ± 0.02	0.03 ± 0.02	
**Neck circumference (cm)**			
**Baseline**	37.36 ± 3.56	36.44 ± 2.86	
**End of trial**	38.0 ± 3.78	35.41 ± 3.03	
***p* ^‡^**	0.010	0.001	
**Change**	−0.64 ± 0.79	1.03 ± 0.96	0.000
**Wrist circumference (cm)**			
**Baseline**	17.93 ± 1.77	17.19 ± 1.67	
**End of trial**	18.43 ± 1.83	16.66 ± 1.67	
***p* ^‡^**	0.001	0.001	
**Change**	−0.50 ± 0.44	0.53 ± 0.50	0.000
**Height/Wrist ratio**			
**Baseline**	9.00 ± 0.99	9.41 ± 0.83	
**End of trial**	8.75 ± 0.93	9.72 ± 0.86	
***p* ^‡^**	0.001	0.001	
**Change**	0.25 ± 0.21	−0.30 ± 0.28	0.000
**Hand grip strength (kg)**			
**Right hand (kg)**			
**Baseline**	12.43 ± 4.94	19.06 ± 5.74	
**End of trial**	9.93 ± 4.05	23.13 ± 6.09	
***p* ^‡^**	0.000	0.000	
**Change**	2.50 ± 1.45	−4.06 ± 2.72	0.000
**Left hand (kg)**			
**Baseline**	13.07 ± 4.80	18.25 ± 6.92	
**End of trial**	9.79 ± 3.79	22.44 ± 6.39	
***p* ^‡^**	0.000	0.000	
**Change**	3.29 ± 1.98	−4.19 ± 2.34	0.000

Data are shown as mean ± standard deviation. Change = baseline–end of trial. *p* ^‡^ as compared between the baseline and end-of-trial for the same group (control or diet); *p* value, as compared between the control and diet groups.

**Table 3:** Biochemical parameters.

Variables	Control (*n* = 14)	Diet (*n* = 16)	*p*
**FPG (mg/dL)**			
**Baseline**	84.50 (76.75–87.00)	88.00 (85.00–96.50)	
**End of trial**	88.50 (81.75–100.0)	90.00 (83.25–94.75)	
***p* ^‡^**	0.197	0.501	
**Change**	−1.00 (−12.50–1.25)	1.00 (−3.00–5.00)	0.077
**CRP (mg/L)**			
**Baseline**	5.27 (2.19–13.33)	4.39 (2.31–7.67)	
**End of trial**	11.50 (4.90–18.57)	2.16 (1.46–3.86)	
***p* ^‡^**	0.002	0.015	
**Change**	−3.63 (−10.28–−1.04)	1.01 (0.03–3.22)	0.000
**ESR (mm/s)**			
**Baseline**	23.00 (9.50–31.00)	31.00 (16.50–51.25)	
**End of trial**	33.00 (13.75–39.50)	25.00 (9.50–39.00)	
***p* ^‡^**	0.001	0.001	
**Change**	−4.00 (−9.75–−2.50)	5.00 (3.00–8.00)	0.000
**AST (µ/L)**			
**Baseline**	20.00 (14.50–21.75)	18.00 (15.00–22.00)	
**End of trial**	18.00 (14.75–24.25)	20.50 (14.00–22.00)	
***p* ^‡^**	0.728	0.362	
**Change**	−1.00 (−3.50–2.25)	−0.50 (−3.75–2.00)	0.918
**ALT (µ/L)**			
**Baseline**	17.00 (10.25–25.25)	17.00 (12.50–21.00)	
**End of trial**	16.50 (11.50–30.00)	17.00 (11.75–22.75)	
***p* ^‡^**	0.484	0.706	
**Change**	−0.05 (−6.25–3.00)	−0.50 (−4.50–3.00)	0.854
**Uric acid (mg/dL)**			
**Baseline**	4.70 (4.38–5.73)	3.70 (3.43–4.18)	
**End of trial**	4.70 (4.15–5.50)	3.40 (2.80–4.45)	
***p* ^‡^**	0.875	0.038	
**Change**	−0.16 (−0.52–0.55)	0.30 (−0.05–0.48)	0.208
**Creatinine (mg/dL)**			
**Baseline**	0.73 (0.65–0.88)	0.66 (0.58–0.76)	
**End of trial**	0.71 (0.63–0.86)	0.62 (0.58–0.73)	
***p* ^‡^**	0.363	0.080	
**Change**	0.02 (−0.03–0.06)	0.01 (−0.01–0.07)	0.667
**Triglycerides (mg/dL)**			
**Baseline**	125.00 (97.00–151.25)	94.50 (76.25–140.25)	
**End of trial**	144.50 (113.75–178.00)	106.00 (83.00–128.50)	
***p* ^‡^**	0.330	0.796	
**Change**	−9.00 (−54.25–24.25)	8.00 (−27.25–18.75)	0.334
**LDL (mg/dL)**			
**Baseline**	116.05 (77.38–132.35)	119.90 (92.73–143.55)	
**End of rial**	124.50 (106.60–141.85)	112.40 (98.58–143.80)	
***p* ^‡^**	0.033	0.352	
**Change**	−22.00 (−41.50–10.78)	5.30 (−7.03–16.05)	0.013
**HDL (mg/dL)**			
**Baseline**	55.55 (44.60–67.35)	52.90 (46.93–68.03)	
**End of trial**	54.95 (45.30–62.50)	53.20 (46.90–64.70)	
***p* ^‡^**	0.258	0.408	
**Change**	2.40 (−5.90–8.83)	0.55 (−3.20–5.08)	0.951
**Total Cholesterol (mg/dL)**			
**Baseline**	186.50 (158.75–214.75)	198.50 (163.75–246.25)	
**End of trial**	215.50 (175.75–232.550)	197.00 (156.00–236.50)	
***p* ^‡^**	0.011	0.255	
**Change**	−22.50 (−41.00–−2.00)	7.50 (−8.25–27.00)	0.008

Data are expressed as median (25th–75th percentile). Change: baseline–end of trial. *p* ^‡^ as compared between the baseline and end of trial for the same group (control or diet); *p* value, as compared between the placebo and synbiotic groups.

**Table 4:** Disease activity.

Variables	Control (*n* = 14)	Diet (*n* = 16)	*p*
**Tender joints**			
**Baseline**	5.57 ± 4.72	5.69 ± 4.64	
**End of trial**	11.50 ± 6.36	1.94 ± 2.74	
***p* ^‡^**	0.000	0.000	
**Change**	−5.93 ± 3.69	3.75 ± 2.35	0.000
**Swollen joints**			
**Baseline**	0.00 (0.00–0.00)	0.00 (0.00–0.00)	
**End of trial**	0.50 (0.00–2.00)	0.00 (0.00–0.00)	
***p* ^‡^**	0.023	0.102	
**Change**	0.00 (−1.00–0.00)	0.00 (0.00–0.00)	0.012
**DAS28–ESR**			
**Baseline**	3.59 ± 1.04	4.68 ± 1.14	
**End of trial**	5.39 ± 0.77	3.01 ± 0.92	
***p* ^‡^**	0.000	0.000	
**Change**	−1.80 ± 0.54	1.68 ± 0.74	0.000
**DAS28–CRP**			
**Baseline**	3.17 ± 0.81	3.80 ± 1.04	
**End of trial**	4.91 ± 0.51	2.15 ± 0.65	
***p* ^‡^**	0.000	0.000	
**Change**	−1.74 ± 0.50	1.66 ± 0.73	0.000
**SDAI**			
**Baseline**	15.31 ± 8.40	20.96 ± 6.93	
**End of trial**	29.69 ± 9.05	11.18 ± 12.63	
***p* ^‡^**	0.000	0.008	
**Change**	−14.39 ± 4.09	9.78 ± 12.83	0.000

Data are presented as mean ± standard deviation and median (25–75th percentile). Change: baseline–end of trial. *p* ^‡^ as compared between the baseline and end of trial for the same group (control or diet); *p* value, as compared between the placebo and synbiotic groups.

## Data Availability

Authors agree to participate in making all data available, if requested.

## Supplementary Materials

The following supporting information can be downloaded at https://www.mdpi.com/article/10.3390/life15030463/s1. Figure S1. Taxonomic analysis of microbiomes: Family levels, Figure S2. Taxonomic analysis of microbiomes: Order levels and Figure S3. Taxonomic analysis of microbiomes: Class levels.

## Abbreviations

The following abbreviations are used in this manuscript:

RA	Rheumatoid arthritis
Th17	T helper 17
SCFA	Short-chain fatty acid
DAS28-ESR	Disease Activity Score-28 Erythrocyte Sedimentation Rate
DAS28-CR	Disease Activity Score-28-C Reactive Protein
SDAI	Simple Disease Activity Index
EULAR	European League Against Rheumatism
ACR	American College of Rheumatology
DMARD	Disease-modifying antirheumatic drug
BMI	Body mass index
NSAIDs	Non-Steroidal Anti-inflammatory Drugs
BIA	Bioelectrical impedance analysis
AETD	American Association of Hand Therapists
FPG	Fasting plasma glucose
LDL	Low-density lipoprotein
HDL	High-density lipoprotein
AST	Aspartate aminotransferase
ALT	Alanine aminotransferase
DNA	Deoxyribonucleic Acid
dsDNA	double-stranded Deoksiribo Nükleik Asit
rRNA	ribosomal Ribo Nucleic Acid
ASVs	Amplicon Sequence Variants
MACs	Microbiota-accessible carbohydrates
