# Supplementary material for: The Effect of the Ideal Food Pyramid on Gut Microbiota in Rheumatoid Arthritis Patients
Source: Life (Basel). 2025 Mar 14;15(3):463. doi: 10.3390/life15030463 (PMC11943791; doi:10.3390/life15030463)

life

MDPI

Supplementary Materials: Figure S1. Taxonomic analysis of microbiomes: Family levels, **Figure S2**. Taxonomic analysis of microbiomes: Order levels, **Figure S3**. Taxonomic analysis of microbiomes: Class levels.

Figure S1. Taxonomic analysis of microbiomes: Family levels

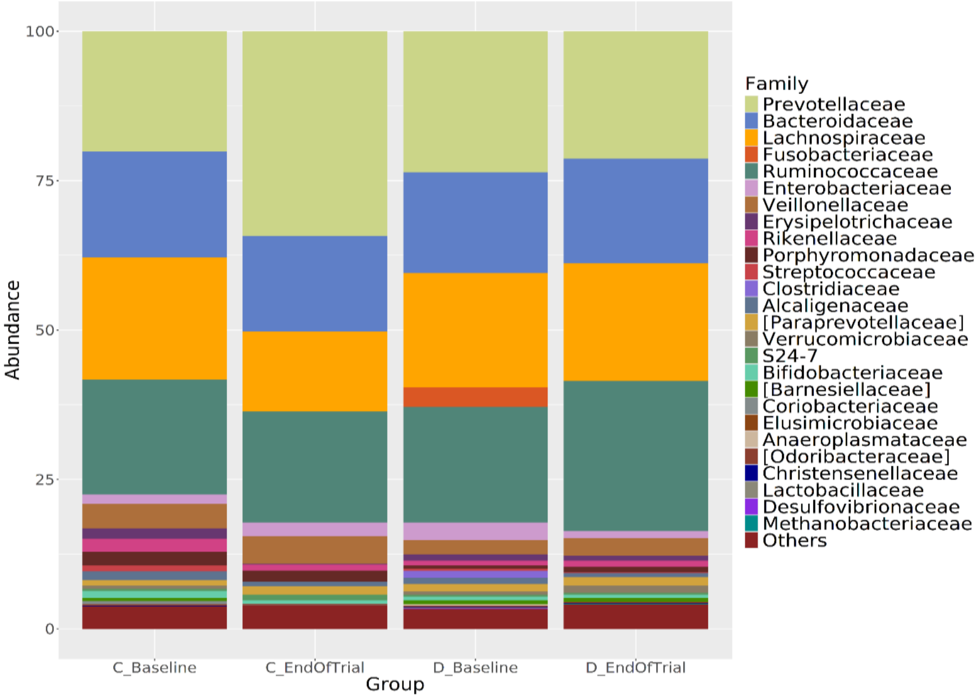

Figure S2. Taxonomic analysis of microbiomes: Order levels

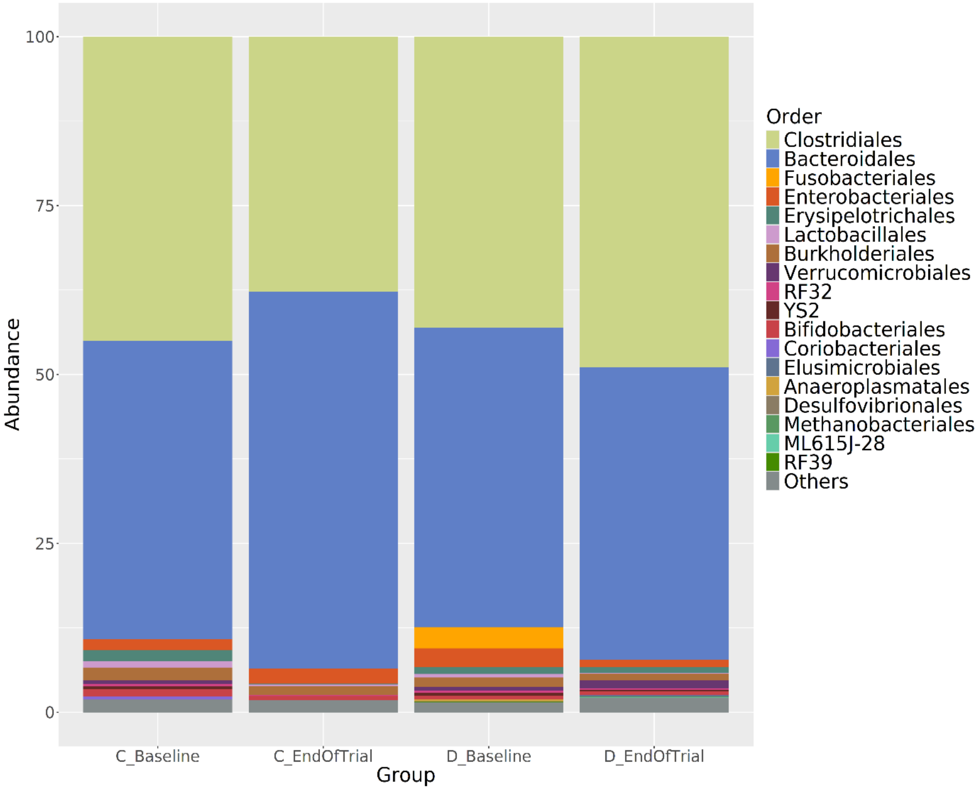

Figure S3. Taxonomic analysis of microbiomes: Class levels

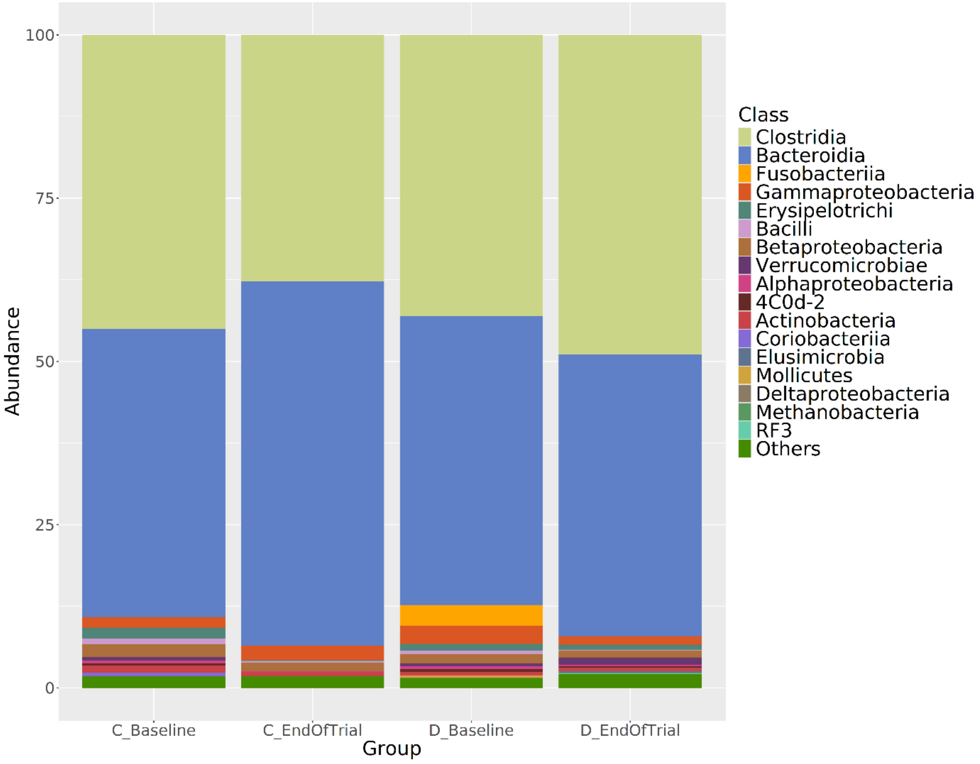

Supplement: Supplementary file 1 [file life-15-00463-s001.zip › life-3462598-supplementary.pdf]
